# Sensory Processing Sensitivity as a Predictor of Proactive Work Behavior and a Moderator of the Job Complexity–Proactive Work Behavior Relationship

**DOI:** 10.3389/fpsyg.2022.859006

**Published:** 2022-08-02

**Authors:** Antje Schmitt

**Affiliations:** Department of Psychology, University of Groningen, Groningen, Netherlands

**Keywords:** job complexity, proactive work behavior, sensory processing sensitivity, employees, person–environment fit

## Abstract

This study investigates the role of sensory processing sensitivity (SPS) as a predictor of employees’ proactive work behavior. SPS is a multidimensional concept that depicts differences in people’s sensory awareness, processing, and reactivity to internal and external influences. Based on research on SPS as grounded in a heightened sensitivity of the behavioral inhibition and activation systems, it was argued that the relationships with task proactivity and personal initiative as indicators of proactive work behavior differ for the three SPS dimensions. Furthermore, based on the person–environment fit perspective, SPS was assumed to moderate the relationship between employees’ job complexity and proactivity. The hypotheses were tested in two two-wave studies (*N* = 215 and *N* = 126). Across both studies, ease of excitation (EOE; i.e., the tendency to be easily overwhelmed by changes) was unrelated to proactivity. Low sensory threshold (LST; i.e., unpleasant arousal from external stimuli) was negatively related to personal initiative, only in Study 2, but it did not predict task proactivity. Meanwhile, aesthetic sensitivity (i.e., AES; awareness of and openness to positive stimuli) was positively related to proactivity, but in Study 2, this relationship could only be established for personal initiative. Moreover, job complexity was positively related to proactivity for those employees high but not for those low in AES. EOE and LST did not act as moderators. This study offers evidence of positive behavioral implications among highly sensitive persons when dealing with job complexity. Overall, the study presents an interesting point of departure for the role of SPS in employee proactivity that calls for more research.

## Introduction

Considering fast-changing work environments and the growth of knowledge-intensive work (Grant and Ashford, 2008; Sung et al., 2017), organizations benefit from employees who engage in proactive work behavior. As a broad term, proactive work behavior denotes individuals’ self-initiated, agentic, and future-oriented efforts to change their work environments or themselves in positive ways (Parker et al., 2010; Parker and Collins, 2010). It entails planning ahead and preparing for anticipated threats and dangers by taking the initiative at present (Frese et al., 1997; Grant and Ashford, 2008). People differ in the extent to which they show proactive work behavior (for an overview, see Wu and Li, 2017). For example, research on the antecedents of proactive work behavior reveals that people high in proactive personality, future orientation, and positive affectivity are more likely to engage in proactivity than those who are low in these traits (Parker et al., 2010; Tornau and Frese, 2013; Wu and Li, 2017).

The first goal of the current study is to add to the stream of research on interindividual differences in proactive work behavior by investigating the relationship between proactive work behavior and the concept of sensory processing sensitivity (SPS). SPS is a specific personality characteristic that captures interindividual differences in people’s awareness and processing of sensory information and their reactivity to internal and external stimuli (Aron and Aron, 1997). It has a strong biological basis, reflected in neurological correlates, and has been examined in human and non-human animals (Greven et al., 2019). SPS has received increasing societal recognition in the last few years (Greven et al., 2019). This is evident from the growth in self-help literature, coaching, and consulting interventions, which are, however, often not based on scientific knowledge (Bröhl et al., 2022). Although research on SPS is growing steadily, studies linking and applying it to employee experiences in the workplace are lacking (exceptions include Andresen et al., 2018; Vander Elst et al., 2019). In particular, little is known about how SPS affects the employees’ self-initiated, future-, and change-oriented work behavior behaviors.

Research proposes that SPS is multidimensional (Smolewska et al., 2006; Lionetti et al., 2019). *Ease of excitation* (EOE) refers to being mentally overwhelmed by internal or external stimuli (e.g., experiencing discomfort when many things occur at once). *Low sensory threshold* (LST) refers to unpleasant sensory arousal in the face of intense stimuli, such as loudness and bright lights. *Aesthetic sensitivity* (AES) relates to the awareness of and openness to positive aspects of one’s surroundings. These three components of SPS are distinctly correlated with other personality traits and individual outcomes, such as well-being (for an overview, see Greven et al., 2019; Lionetti et al., 2019). A deeper cognitive processing of and a stronger reactivity to both positive and negative stimuli might have differential effects on employees’ proactive work behavior. In line with theory and previous research that argues that EOE and LST are associated with heightened activity in the behavioral inhibition system (BIS; Gray, 1991; Pluess et al., 2018), I argue that these dimensions may act as vulnerability factors that inhibit approach behaviors, such as proactivity. AES is assumed to be conducive to proactive work behavior due to its relationship with the heightened sensitivity of the behavioral activation system (BAS; Gray, 1991), indicating high appetitive motivation and the urge to engage in approach behavior.

**Figure 1:**
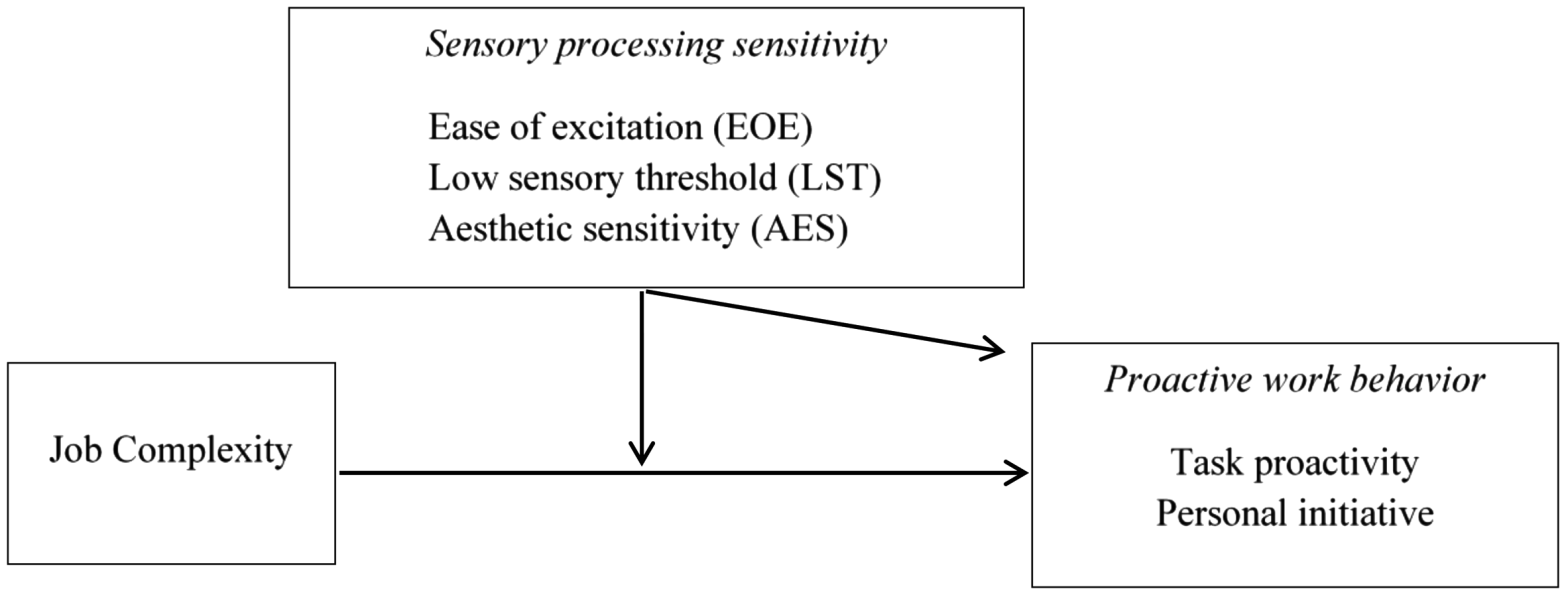
Conceptual model.

The second goal of this study is to investigate whether SPS explains interindividual differences in how employees respond to job complexity in terms of their proactive work behavior. Job complexity refers to a job being mentally demanding, difficult, and challenging to perform (Campbell, 1988; Humphrey et al., 2007). Theory and research widely suggest positive relationships between perceived job complexity and proactive work behavior (Ohly et al., 2006; Frese et al., 2007; Ohly and Schmitt, 2017). However, job complexity might not be seen as desirable by all employees; it can also have costs for the individual because it draws on resources, such as mental energy, potentially causing strain (LePine et al., 2005; Sung et al., 2017). Based on the person–environment (P–E) fit perspective (Kristof-Brown et al., 2005) and the notion that dispositions interact with perceived situational demands to shape proactive work behavior (Wu and Li, 2017), I argue that the relationship between job complexity and proactivity differs depending on employees’ SPS level. Specifically, individuals with high EOE and LST may tend to feel distressed when working under high complexity; for them, high job complexity is likely to be a poor fit, resulting in reduced proactive work behavior. In contrast, individuals with low EOE and LST, who are less sensitive to overstimulation, are more likely to meet the demands of a highly complex job. They may be better able to engage in their work cognitively and perceive complexity as motivating. As a result, the levels of their self-initiated and future-oriented behavior increase. Furthermore, I expect the relationship between job complexity and proactivity to be stronger for individuals with high (versus low) AES, that is, those individuals who show a greater awareness of positive stimuli and are open to approaching new environments. The conceptual model is depicted in Figure 1.

The present study aims to contribute to the research on SPS as a predictor of employees’ proactivity and may specifically inform research about the roles of the different SPS dimensions in employee proactivity. Further, the study adds to our knowledge of the role of job design in proactive work behavior. Studying individual differences in employees’ sensitivity to the environment and the self that can explain when or for whom complex jobs can stimulate proactive work behavior is critical for both research and practice (Sung et al., 2017). Finally, by investigating the factor structure of SPS across two studies, the present research contributes to the ongoing discussion in the SPS literature on its multidimensional conceptualization and measurement (Greven et al., 2019; Hellwig and Roth, 2021).

## Theory and Hypotheses

### SPS and Proactive Work Behavior

Employees may engage in different forms of proactivity, such as making recommendations for work-related changes, preventing the occurrence of problems, or crafting their jobs to establish a better fit with their skills and interests (Parker and Collins, 2010). Here, I focus on two facets of proactive work behavior: individuals’ task proactivity (Griffin et al., 2007) and personal initiative (Frese et al., 1997). Task proactivity is defined as actively initiating changes, such as generating ideas to improve the way in which core tasks are performed (Griffin et al., 2007). Personal initiative is a behavioral style characterized by taking a self-starting approach to work. Employees with high levels of personal initiative anticipate future opportunities, take initiative at work even when others do not, and are persistent in their behaviors (Frese et al., 1997; Frese and Fay, 2001).

Various personality traits have been found to explain interindividual differences in proactive work behaviors (for an overview, see Tornau and Frese, 2013; Wu and Li, 2017). For instance, research has consistently shown that employees high in proactive personality (i.e., general tendency to show initiative, identify opportunities, and act on them to influence one’s environment across situations and times; Bateman and Crant, 1993) are more likely to engage in proactive work behavior. Moreover, individuals’ general disposition of experiencing positive moods and emotions, such as enthusiasm, alertness, and joy, has been found to be positively related to employees’ personal initiative (Den Hartog and Belschak, 2007). Besides, in a sample of 478 German employees, Fay and Frese (2000) found that conservatives (operationalized as individuals with a high intolerance of uncertainty), who are committed to hierarchic values and emphasize the value of traditional practices, show less initiative at work and are less likely to introduce innovations compared to those who are less conservative.

The personality characteristic of SPS—which has mainly been investigated in the neuro-cognitive and developmental psychology literature—causes some people to perceive and process sensory information more thoroughly than others and to be generally more vulnerable to and show higher reactivity to environmental influences (Belsky and Pluess, 2009; Aron et al., 2012). Grounded in the idea of differential susceptibility, SPS is based on the perspective that individuals differ in their susceptibility to stimuli regardless of whether they are exposed to negative or positive influences (Belsky and Pluess, 2009). That is, people high in SPS are more likely than those low in SPS to be adversely affected by negative experiences. At the same time, they may also benefit more from enriching environments, and they are more oriented toward positive stimuli (Vander Elst et al., 2019). Accordingly, SPS was found to be associated with stronger responses to both positive and negative stimuli, such as sad and happy emotional states of others (Acevedo et al., 2014). Thus, being sensitive is not only associated with negative consequences, such as increased risk of stress-related outcomes (e.g., fatigue) or job-related turnover intentions (Evers et al., 2008; Andresen et al., 2018); it may also have positive effects, such as a greater susceptibility to positive social environments and higher learning and creativity (Acevedo et al., 2014; Harms et al., 2019).

The differential positive and negative effects can be attributed to the different subdimensions of SPS (Smolewska et al., 2006) and their distinct underlying motivational processes. Specifically, the dimensions of EOE and LST reflect sensitivity to negative experiences and stimuli. EOE and LST mainly operate through the BIS (Smolewska et al., 2006; Gerstenberg, 2012; Pluess et al., 2018; Lionetti et al., 2019). When the BIS is activated, individuals become more alert, focus their attention on the potentially threatening stimulus or situation, and tend to pause current behavior (Carver and White, 1994). BIS activation is also related to the experience of negative emotions, including anxiety and nervousness (Gray, 1990; Carver and White, 1994; Merchán-Clavellino et al., 2019).

In line with this perspective, I propose that employees high in EOE and LST, who tend to be easily overwhelmed by changes and various stimuli in their environment and thus tend to avoid demanding and potentially threatening situations and risks, are, on average, less likely to show self-initiative behavior that is change-oriented.

Indirect evidence of this can be found in research on neuroticism and proactivity. People high in neuroticism tend to frequently experience aversive cognitive-emotional states, such as anger and threat, and ambiguous and uncertain situations are likely to elicit such negative emotional responses (Watson and Casillas, 2003; Bajcar and Babiak, 2020; Schmitt et al., 2022). Consequently, such individuals might feel uncomfortable initiating potentially risky and change-oriented proactive behavior themselves (Wu and Li, 2017). Accordingly, meta-analytic evidence suggests that neuroticism is negatively correlated with different proactivity concepts, although the relationships are small (Tornau and Frese, 2013; Wu and Li, 2017). Neuroticism is positively associated with SPS (Bröhl et al., 2022), particularly with the dimensions of EOE and LST (Lionetti et al., 2019; Hellwig and Roth, 2021), and it is consistently found to relate to BIS activity (Carver and White, 1994).

*Hypothesis 1a*: EOE and LST are negatively related to employees’ proactive work behavior.

AES was found to have different patterns of relationships with individual outcomes than EOE and LST. AES is related positively to self-efficacy and attention to detail (Evers et al., 2008; Greven et al., 2019), and it is positively related to the sensitivity of the BAS (Gray, 1991; McNaughton and Gray, 2000; Lionetti et al., 2019). BAS activation was found to predict activating behaviors, such as entrepreneurial action (Lerner et al., 2018), and students’ study engagement and academic performance (van Beek et al., 2013). AES further shares some aspects with and is moderately to strongly related to the Big Five trait openness to experiences (Listou Grimen and Diseth, 2016; Lionetti et al., 2019; Hellwig and Roth, 2021; Bröhl et al., 2022). Both AES and openness to experiences are characterized by people’s tendency to seek out positive and stimulating environments. In their meta-analysis, Tornau and Frese (2013) found positive but small relationships between openness to experiences and proactivity concepts. Based on these perspectives, I propose that employees high in AES, who are curious, imaginative, broad-minded, more sensitive to positive aspects in their environment, and appreciate new experiences and changes, are more likely to show proactive work behaviors than those low in AES.

*Hypothesis 1b*: AES is positively related to proactive work behavior.

### SPS as a Moderator of the Relationship Between Job Complexity and Proactive Work Behavior

Apart from investigating the relationships between personality traits and proactivity, scholars have, based on job design and job enrichment frameworks (Humphrey et al., 2007; Parker, 2017), considered various job characteristics as antecedents of proactive work behavior (for an overview, see Ohly and Schmitt, 2017). Complexity has been identified as an important knowledge characteristic of jobs (Morgeson and Humphrey, 2006; Humphrey et al., 2007). It refers to the level to which work tasks are multifaceted and difficult to perform for the individual. Jobs high in complexity are likely to include tasks characterized by ambiguity and conflicting elements that require the use of diverse and complex skills and are mentally challenging (Campbell, 1988; Humphrey et al., 2007). The literature distinguishes between job demands that primarily hinder individuals and those that challenge them (LePine et al., 2005). It is argued that job complexity is typically appraised as a challenge demand, a positive-motivational aspect of one’s work that may promote psychological empowerment, learning, job satisfaction, and the stimulation of creative ideas (Shalley et al., 2004; Morgeson and Humphrey, 2006; Frese et al., 2007). Working on complex tasks steers attention, activates effort, and provides opportunities for proactivity. Accordingly, job complexity was found to be positively related to different forms of employees’ proactivity, such as their personal initiative and suggestion-making at work (e.g., Frese et al., 2007; for an overview, see Ohly et al., 2006; Ohly and Schmitt, 2017). Thus, following previous literature, I propose the following hypothesis.

*Hypothesis 2*: Job complexity is positively related to proactive work behavior.

Despite the generally positive relationships between job complexity and outcomes, such as proactivity, evidence shows that these positive effects do not hold under all conditions. Rather, the relationships between job characteristics, such as job complexity and employee proactivity, may be more complex. Working on complex tasks requires a high level of cognitive information processing from the individual, including high attentional control and cognitive flexibility (Chen et al., 2001), which can be burdensome and may result in cognitive overload for some people (Humphrey et al., 2007; Sung et al., 2017). Accordingly, some evidence suggests that job complexity is positively related to emotional exhaustion and job-related anxiety in employees (Xie and Johns, 1995; De Jonge and Schaufeli, 1998).

The current study builds on the idea that research should focus on the interplay of perceived job or situational demands and dispositional factors to predict employees’ proactive behavior at work (Wu and Li, 2017). I assume that individuals with high (versus low) SPS perceive and manage job complexity differently. Based on the P–E fit approach (Kristof-Brown et al., 2005), I argue that SPS acts as a boundary condition of the relationship between job complexity and employees’ proactivity. P–E fit is defined as the “compatibility between an individual and a work environment that occurs when their characteristics are well matched” (Kristof-Brown et al., 2005, p. 281). The correspondence between individuals’ attributes and characteristics of their environment may affect their motivation, behavior, and well-being (Kristof-Brown et al., 2005; Schmitt et al., 2015). Accordingly, the relationship between job complexity and individuals’ proactive work behavior depends on the level of fit with their personality characteristics. The three SPS dimensions represent different proxies of fit and may thus have varying effects on how employees manage job complexity regarding their proactivity.

#### EOE and LST as Moderators

Based on the P–E fit perspective, for individuals high in EOE and LST, who are sensitive to potentially threatening stimuli and are easily overwhelmed, high job complexity might represent a misfit between person and environment that can lead to behavioral inhibition and self-regulation to prevent over-arousal (Andresen et al., 2018). By focusing on complex work tasks, individuals high in EOE and LST may have fewer cognitive resources (e.g., vigilance and attention) available to engage in self-initiated, future-, and change-oriented behavior. Furthermore, as BIS activity inhibits action toward goals, individuals’ engagement in self-initiated and change-oriented goals may suffer (Sobocko and Zelenski, 2015). In contrast, employees with low EOE and LST, who are not easily overwhelmed by a complex and ambiguous environment, have more available resources to exceed the minimum requirements (Schmitt et al., 2016; Sung et al., 2017). For them, complexity is more likely to be experienced as motivating. Altogether, I argue that when faced with increasing job complexity, employees with high EOE and LST may respond less favorably than those with low EOE and LST regarding their proactive work behavior.

*Hypothesis 3a*: EOE and LST moderate the relationship between job complexity and proactive work behavior. Specifically, the positive relationship is stronger for individuals with low EOE and LST and is less strong or negative for individuals with high EOE and LST.

### AES as Moderator

Individuals with high AES, who are aware of nuances and appreciate positive aspects in their surroundings, are attentive to details and have a high level of imagination and openness to positive experiences. When faced with job complexity, they may be more likely to see beyond the current circumstances and envision proactive changes. Furthermore, working on complex tasks activates attention, and compared with individuals low in AES, those with high AES may be more likely to use this attention to explore new possibilities and alternative courses of action and create new ideas for performing tasks more effectively and efficiently (Shalley et al., 2004). Similarly, Espedido and Searle (2020) argued that individuals high in BAS, who approach situations that have the potential for personal growth or mastery, tend to appraise those situations as being more challenging and motivating.

For employees who perceive their jobs as complex, those with high AES may be likely to engage in proactivity and accept opportunities to change things for improvement. Accordingly, AES may strengthen the relationship between job complexity and employee proactivity.

*Hypothesis 3b*: AES moderates the relationship between job complexity and proactive work behavior. Specifically, the positive relationship is stronger for individuals with high AES, and it is less strong for individuals with low AES.

## Overview of the Present Studies

The hypotheses were tested in two two-wave studies. Specifically, Hypotheses 1a and 1b were tested in Study 1, while Study 2 aimed to also test Hypotheses 2, 3a, and 3b. Both studies had a time lag of one month between the measurements of the predictors and moderators and the outcomes to prevent common method bias (Podsakoff et al., 2003). Study 1 examines the main effects of the SPS dimensions on task proactivity and personal initiative as two relevant indicators of proactive employee work behavior. In Study 2, the job complexity–proactive work behavior relationship and the moderating role of the SPS dimensions is tested using the same two proactivity indicators as in Study 1. Across both studies, a shortened measurement for SPS is used (Listou Grimen and Diseth, 2016), which has been developed in the wake of criticism of the original scale by Aron and Aron (1997). Using this shortened scale, the study investigates whether the three-dimensional structure of SPS holds across both studies.

The data for both studies were gathered as part of larger projects. Therefore, the questionnaires included several measures that are irrelevant to and not described in the present paper. No other studies based on these two datasets have been published. Both studies were approved by the ethics committee of the Department of Psychology at the University of Groningen, the Netherlands.

## Study 1

### Method

#### Procedure and Sample

The study was conducted in January and February 2020. The research-focused crowd-working platform Prolific was used to recruit the study participants (Peer et al., 2017; Palan and Schitter, 2018). Two hundred eighty-three out of 300 individuals completed the time 1 (T1) survey (response rate 94.3%). Among them, 27 failed at least one out of three attention check items and were excluded from the sample. The others (*N* = 256) were approached 1 month later and asked to complete the time 2 (T2) survey. Two hundred thirty-four individuals participated. Some participants provided missing data on the core study variables and were excluded (*N* = 19). The final sample of individuals who participated in both waves was 215. Attrition analyses revealed that individuals who participated in both waves were older than the incomplete responders who dropped at T2 [*t*(74.275) = −2.80, *p* = 0.007].

Of the participants, 103 (47.9%) were female. Participants’ mean age was 33.63 years (range between 19 and 64 years). Fifty-five (25.6%) were from the United Kingdom, and the remainder was from various countries, such as the United States, Canada, Ireland, Poland, Italy, Spain, and Greece. Regarding their highest level of education, 64 (29.8%) attained a high school degree, and 151 (70.2%) had a university degree. The participants had different professional backgrounds, such as business analyst, dentist, carpenter, IT manager, journalist, nurse, sales assistant, secretary. Their average organizational tenure was 5.58 years (*SD* = 5.79).

#### Measures

At T1, the participants provided information about their demographics and their SPS. At T2, they were asked to rate their proactive work behavior as shown across the past weeks. The survey items were presented in English.

##### Sensory Processing Sensitivity

The 13 item scale by Listou Grimen and Diseth (2016), a shortened and validated version of the original 27 item scale (Aron and Aron, 1997), was used to measure the three SPS dimensions. The participants were asked to indicate their level of agreement with items describing various aspects of sensitivity relating to their feeling and reactions to internal and external stimuli on a five-point scale ranging from 1 *(does not apply at all)* to 5 *(fully applies)*. EOE was measured by five items (Cronbach’s alpha = 0.73). An example item is “I am annoyed when people try to get me to do too many things at once.” LST consisted of three items (alpha = 0.74), e.g., “I am easily overwhelmed by things like bright light, strong smells, coarse fabric, or sirens close by.” AES was assessed by five items (alpha = 0.67) with the example item: “I enjoy delicate or fine scents, tastes, sounds, works of arts.” Confirmatory factor analysis (CFA) in Mplus 8.1 (Muthén and Muthén, 1998-2017) was conducted to test the factorial validity of the scale. The fit indices of the three-dimensional model were *χ*^2^(62) = 124.917, CFI = 0.893; RMSEA = 0.069, SRMR = 0.064. These indices were slightly below the accepted criteria for cut-off values (Hu and Bentler, 1998), but they were superior to the other possible (Aron and Aron, 1997; Evans and Rothbart, 2008) and more parsimonious models with one factor [*χ*^2^(65) = 321.013, CFI = 0.566; RMSEA = 0.135, SRMR = 0.114] and two factors [*χ*^2^(64) = 179.081, CFI = 0.805; RMSEA = 0.091, SRMR = 0.073; EOS and LST as one factor].

##### Proactive Work Behavior

Participants were asked to refer to their work behaviors during the past few weeks. Three items from a scale by Griffin et al. (2007) were used to measure task proactivity. An example item is “I initiated better ways of doing my core tasks.” Responses ranged from 1 *(does not apply at all)* to 5 *(fully applies)*. Cronbach’s alpha of the scale was 0.90. Personal initiative was assessed with five items by Spychala and Sonnentag (2011, based on Frese et al., 1997). An example item is “I took matters into my own hands at work.” The participants provided their responses on five-point scales ranging from 1 *(strongly disagree)* to 5 *(strongly agree)*. Cronbach’s alpha was 0.77.

##### Control Variables

In line with previous research (e.g., Bolino and Turnley, 2005; Hong et al., 2016; Mensmann and Frese, 2019), the effects of participants’ age and gender were statistically controlled. Research reveals that proactive work behavior might change with age (Frese and Fay, 2001; Thomas et al., 2010). Gender has been shown to correlate with proactive work behavior with males engaging in higher levels (e.g., Bolino and Turnley, 2005; Griffin et al., 2007), albeit correlations are small (Tornau and Frese, 2013).

### Data Analysis and Results

Descriptive statistics and correlations among the study variables are presented in Table 1. EOE and LST at T1 were positively and significantly related (*r* = 0.47, *p* < 0.01), but AES T1 was unrelated to both EOE T1 (*r* = −0.01, *ns*) and LST T1 (*r* = 0.10, *ns*). While AES T1 was positively related to task proactivity at T2 (*r* = 0.26, *p* < 0.01), and personal initiative at T2 (*r* = 0.28, *p* < 0.01), LST and EOE at T1 were unrelated to both indicators of proactivity, as reported 1 month later. Hierarchical multiple regression analyses were used to test Hypotheses 1a and 1b. The control variables age and gender were entered in the first step and EOE, LST, and AES T1 were entered in the second step to predict task proactivity and personal initiative at T2, respectively. The variance inflation factors (VIF) were inspected to estimate the degree of collinearity among the variables in the regression analysis. All VIF scores were below 2. Specifically, they ranged between 1.09 for the effect of age on task proactivity at T2 and 1.76 for the effect of LST T1 on task proactivity at T2. This indicates that multicollinearity was not a serious threat (Chatterjee and Price, 1991).

**Table 1:** Means (M), SD, and correlations of the study variables in Study 1.

		*M*	*SD*	1	2	3	4	5	6
1	Age T1	33.63	9.61	−					
2	Gender T1	0.52	0.50	−0.03	−				
3	EOE T1	3.46	0.77	−0.11	0.11	−			
4	LST T1	2.97	1.00	−0.10	0.08	0.47^**^	−		
5	AES T1	3.58	0.66	0.05	−0.13	−0.01	0.10	−	
6	Task proactivity T2	3.40	0.93	−0.05	−0.05	0.04	−0.00	0.26^**^	−
7	Personal initiative T2	3.65	0.67	0.05	−0.09	−0.05	0.04	0.28^**^	0.52^**^

*N* = 215. T1 = time 1; T2 = time 2. EOE, ease of excitation; LST, low sensory threshold; and AES, aesthetic sensitivity. Gender was coded 0 = female, 1 = male.

** *p* ≤ 0.01.

Hypothesis 1a states that EOE and LST are negatively related to employee proactive work behavior. Both EOE T1 (*β* = 0.07, *p* = 0.380) and LST T1 (*β* = −0.06, *p* = 0.411) were unrelated to task proactivity at T2 (see Table 2) and to personal initiative at T2 (EOE: *β* = −0.01, *p* = 0.916; LST: *β* = −0.06, *p* = 0.410, see Table 3) with the control variables included. These results do not support Hypothesis 1a.

**Table 2:** Results of the hierarchical multiple regression analysis with task proactivity at T2 as dependent variable (Study 1).

Variable	Task proactivity T2					
	*B*	*SE*	*β*	*B*	*SE*	*β*
**Control variables**						
Age T1	−0.01	0.01	−0.05	−0.01	0.01	−0.06
Gender T1	−0.09	0.13	−0.05	−0.03	0.13	−0.02
**Main effects**						
EOE T1				0.08	0.09	0.07
LST T1				−0.06	0.07	−0.06
AES T1				0.38	0.10	0.27^**^
*R* ^2^	0.005			0.076		

*N* = 215. T1 = time 1; T2 = time 2. EOE, ease of excitation; LST, low sensory threshold; and AES, aesthetic sensitivity. *R*^2^ = proportion of variance explained in the criterion.

** *p* ≤ 0.01.

**Table 3:** Results of the hierarchical multiple regression analysis with personal initiative at T2 as dependent variable (Study 1).

Variable	Personal initiative T2					
	*B*	*SE*	*β*	*B*	*SE*	*β*
**Control variables**						
Age T1	0.00	0.01	0.04	0.00	0.01	0.02
Gender T1	−0.11	0.09	−0.08	−0.06	0.09	−0.04
**Main effects**						
EOE T1				−0.01	0.07	−0.01
LST T1				−0.04	0.05	−0.06
AES T1				0.29	0.07	0.28^**^
*R* ^2^	0.009			0.087		

*N* = 215. T1 = time 1, T2 = time 2. EOE, ease of excitation; LST, low sensory threshold; and AES, aesthetic sensitivity. *R*^2^ = proportion of variance explained in the criterion.

** *p* < 0.01.

Hypothesis 1b states that AES positively predicts employees’ proactive work behavior. The results showed that AES T1 was significantly and positively associated with task proactivity at T2 (*β* = 0.27, *p* < 0.001) and with personal initiative at T2 (*β* = 0.28, *p* < 0.001), supporting Hypothesis 1b. By adding the three SPS dimensions, 7.1% additional variance in task proactivity and 7.8% in personal initiative was explained above and beyond the control variables. All results were equivalent when the control variables were excluded from the analyses, which indicates that the control variables do not affect or provide alternative explanations for the relationships between the independent and dependent variables.

## Study 2

### Method

#### Procedure and Sample

Study 2 aimed to replicate the results from Study 1 and to further test if EOE, LST, and AES moderate the positive relationship between job complexity and proactive work behaviors. The study was conducted in August and September 2020 and Prolific was used to recruit the study participants. At T1, 200 individuals (all English native speakers) were invited to take part in the study and 191 of those completed the survey (95.5% response rate). All participants from the first wave were contacted again 1 month later asking them to complete the T2 survey. One hundred twenty-six participants responded. The final sample consisted of these 126 individuals who participated in both waves. Differences in the study variables and core demographic variables were calculated between participants who provided data only at T1 and the complete responders. Participants who provided incomplete data had higher LST scores than those who participated in both waves [*t*(114.974) = 2.171, *p* = 0.032] indicating that LST is associated with a higher likelihood of dropping out.

Participants’ mean age at T1 was 34.61 years (*SD* = 9.52) and women made up 65.9% of the sample. Most participants (92.1%) were living in the United Kingdom and 7.9% came from other English-speaking countries such as Ireland, Canada, and the United States. In terms of their educational level, 48 (38.1% of the sample) had received a high school degree and 78 (61.9%) held a university degree. The participants represented a variety of occupations (e.g., accountant, financial manager, social worker, teacher, web designer, receptionist). Their average organizational tenure was 6.13 years (*SD* = 5.08).

#### Measures

At T1, the participants provided information about their demographics, their general SPS, and their perceived level of job complexity referring to the past few weeks. At T2, they were asked to rate their proactive work behavior during the last few weeks. The survey items were presented in English.

##### Sensory Processing Sensitivity

The same 13 items as in Study 1 were used to measure SPS (Listou Grimen and Diseth, 2016). Cronbach’s alphas were 0.70 for the five-item EOE scale, 0.90 for LST, and 0.77 for AES. I examined the measurement model of the SPS construct using CFA. Results showed that a three-factor-model yielded a good fit to data [*χ*^2^(62) = 85.170, CFI = 0.959, RMSEA = 0.054. SRMR = 0.064] and fit the data significantly better than a one-factor [*χ*^2^(65) = 332.527, CFI = 0.530, RMSEA = 0.181, and SRMR = 0.166] and a two-factor model with EOE and LST forming one factor [*χ*^2^(64) = 170.140, CFI = 0.813, RMSEA = 0.115, and SRMR = 0.108].

##### Job Complexity

The four-item scale developed by Maynard and Hakel (1997) was used to measure job complexity at T1. An example item is: “My work tasks were mentally demanding.” The response format ranged from 1 *(strongly disagree)* to 5 *(strongly agree)*. Cronbach’s alpha was 0.86.

##### Proactive Work Behavior

Task proactivity was measured with the same three items as in Study 1 (Griffin et al., 2007). Cronbach’s alpha was 0.91. The seven-item scale by Frese et al. (1997) was used to measure personal initiative. Participants were asked to indicate to what extent they showed initiative at work in the past few weeks. An example item is: “I used opportunities quickly in order to attain my goals.” Participants provided their responses on a five-point scale (1 *= does not apply at all* to 5 = *fully applies*). Cronbach’s alpha was 0.85.

##### Control Variables

As in Study 1, participants’ age and gender were included as control variables (Hong et al., 2016; Mensmann and Frese, 2019).

### Data Analysis and Results

Descriptive statistics and correlations among all variables are shown in Table 4. LST and EOE at T1 were significantly related (*r* = 0.39, *p* < 0.01), but AES T1 was unrelated to EOE T1 (*r* = −0.15, ns) and LST T1 (*r* = 0.06, ns). Job complexity T1 was significantly related to personal initiative at T2 (*r* = 0.23, *p* < 0.01), but the relationship with task proactivity T2 was not significant (*r* = 0.13, ns). Hierarchical multiple regression analysis was carried out to test the hypotheses. I mean-centered the variables before calculating the interaction terms for job complexity T1 and each of the three SPS dimensions (Cohen et al., 2003). Variables were then entered in three steps: The control variables gender and age were entered in the first step and the linear main effects of job complexity and the three SPS variables were entered in the second step. Finally, in the third step, the linear interaction terms were entered for each of the three SPS dimensions separately (see Tables 5 and 6). The VIF values across all analyses were below 2 with the highest score of 1.38 for the effect of EOE T1 on personal initiative T2.

**Table 4:** Means (M), SD, and correlations of the study variables in Study 2.

		*M*	*SD*	1	2	3	4	5	6	7
1	Age T1	34.61	9.52	−						
2	Gender T1	0.34	0.48	0.01	−					
3	EOE T1	3.31	0.78	−0.25^**^	−0.18^*^	−				
4	LST T1	1.93	1.03	−0.02	−0.07	0.39^**^	−			
5	AES T1	3.15	0.80	−0.02	−0.04	−0.15	0.06	−		
6	Job complexity T1	3.71	1.00	0.03	−0.04	−0.01	0.00	0.06	−	
7	Task proactivity T2	3.17	1.00	−0.17	−0.05	0.01	−0.04	0.14	0.13	−
8	Personal initiative T2	3.68	0.65	0.03	−0.14	−0.15	−0.22^*^	0.21^*^	0.23^**^	0.59^**^

*N* = 126. T1 = time 1, T2 = time 2. EOE, ease of excitation; LST, low sensory threshold; and AES, aesthetic sensitivity. Gender was coded 0 = female, 1 = male.

* *p* ≤ 0.05;

** *p* ≤ 0.01.

**Table 5:** Results of hierarchical multiple regression analysis with task proactivity at T2 as Dependent Variable (Study 2).

	Dependent variable: task proactivity T2								
	EOE as moderator			LST as moderator			AES as moderator		
	*B*	*SE*	*β*	*B*	*SE*	*β*	*B*	*SE*	*β*
**Step 1: controls**									
Age	−0.02	0.01	−0.17	−0.02	0.01	−0.17	−0.02	0.01	−0.17
Gender	−0.10	0.19	−0.05	−0.10	0.19	−0.05	−0.10	0.19	−0.05
*R* ^2^	0.031			0.031			0.031		
**Step 2: main effects**									
Job complexity T1	0.12	0.09	0.12	0.12	0.09	0.12	0.12	0.09	0.12
EOE T1	0.00	0.13	0.00	0.00	0.13	0.00	0.00	0.13	0.00
LST T1	−0.06	0.10	−0.06	−0.06	0.10	−0.06	−0.06	0.10	−0.06
AES T1	0.17	0.12	0.13	0.17	0.12	0.13	0.17	0.12	0.13
*R* ^2^	0.068			0.068			0.068		
**Step 3: interaction**									
Job complexity T1 * EOE T1	−0.21	0.12	−0.16						
Job complexity T1 * LST T1				0.16	0.09	0.16			
Job complexity T1 * AES T1							0.26	0.11	0.22^*^
*R* ^2^	0.090			0.092			0.114		

*N* = 126. T1 = time 1, T2 = time 2. Gender was coded 0 = male, 1 = female. The predictors were mean-centered. B, unstandardized regression coefficient and SE, standard error. *R*^2^ = proportion of variance explained in the criterion.

* *p* ≤ 0.05.

**Table 6:** Results of hierarchical multiple regression analysis with personal initiative at T2 as dependent variable (Study 2).

	Dependent variable: personal initiative T2								
	EOE as moderator			LST as moderator			AES as moderator		
	*B*	*SE*	*β*	*B*	*SE*	*β*	*B*	*SE*	*β*
**Step 1: controls**									
Age	0.00	0.01	0.03	0.00	0.01	0.03	0.00	0.01	0.03
Gender	−0.19	0.12	−0.14	−0.19	0.12	−0.14	−0.19	0.12	−0.14
*R* ^2^	0.022			0.022			0.022		
**Step 2: main effects**									
Job complexity T1	0.14	0.05	0.21^*^	0.14	0.05	0.21^*^	0.14	0.05	0.21^*^
EOE T1	−0.04	0.08	−0.05	−0.04	0.08	−0.05	−0.04	0.08	−0.05
LST T1	−0.14	0.06	−0.22^*^	−0.14	0.06	−0.22^*^	−0.14	0.06	−0.22^*^
AES T1	0.16	0.07	0.20^*^	0.16	0.07	0.20^*^	0.16	0.07	0.20^*^
*R* ^2^	0.170			0.170			0.170		
**Step 3: interaction**									
Job complexity T1 * EOE T1	−0.09	0.07	−0.11						
Job complexity T1 * LST T1				0.05	0.06	0.08			
Job complexity T1 * AES T1							0.14	0.07	0.18^*^
*R* ^2^	0.181			0.175			0.201		

*N* = 126. T1 = time 1, T2 = time 2. Gender was coded 0 = male, 1 = female. The predictors were mean-centered. B, unstandardized regression coefficient and SE, standard error. *R*^2^ = proportion of variance explained in the criterion associated by the variables.

* *p* ≤ 0.05.

Both EOE T1 and LST T1 were unrelated to employee task proactivity at T2 (*β* = 0.00, *p* = 0.973 for EOE and *β* = −0.06, *p* = 0.552 for LST). EOE T1 also did not predict personal initiative at T2 (*β* = −0.05, *p* = 0.623) whereas LST predicted personal initiative at T2 negatively (*β* = −0.22, *p* = 0.018). Hypothesis 1a is only partially supported for LST T1 predicting personal initiative at T2. AES T1 did not predict task proactivity at T2 (*β* = 0.13, *p* = 0.131), but it predicted personal initiative T2 (*β* = 0.20, *p* = 0.017). Hypothesis 1b is thus partially supported. Job complexity T1 did not predict task proactivity significantly (*β* = 0.12, *p* = 0.167), but it was positively and significantly related to personal initiative at T2 (*β* = 0.21, *p* = 0.012). This partially supports Hypothesis 2.

Hypothesis 3a states that the relationship between job complexity and proactive work behavior is stronger for individuals low in EOE and LST and less strong or negative for individuals high in EOE and LST. The results are shown in Tables 5 and 6. The interaction terms were non-significant for task proactivity at T2 (*β* = −0.16, *p* = 0.089 for EOE and *β* = 0.16, *p* = 0.077 for LST) and for personal initiative at T2 (*β* = −0.11, *p* = 0.216 for EOE and *β* = 0.08, *p* = 0.387 for LST). Thus, Hypothesis 3a was not supported by the data.

**Figure 2:**
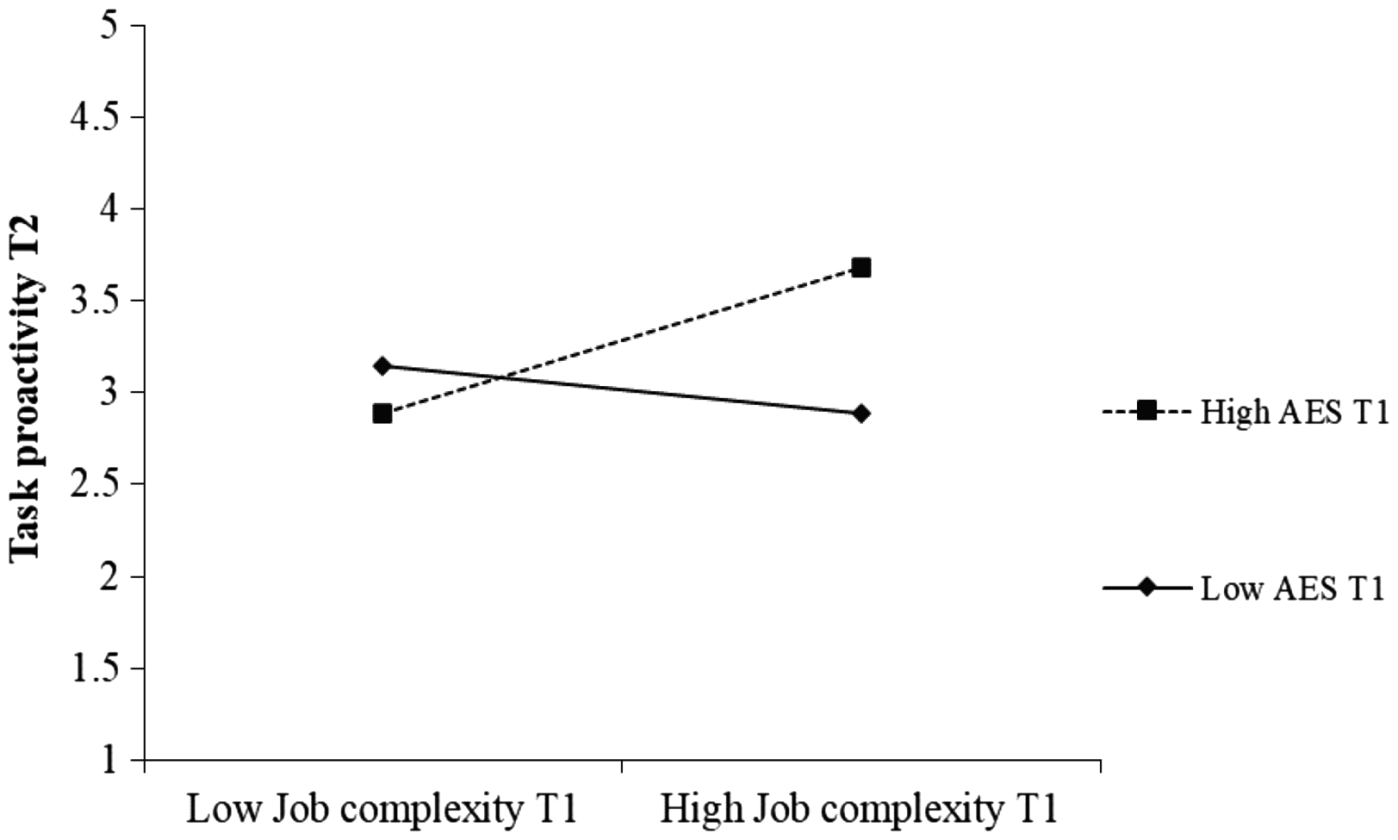
The moderating effect of aesthetic sensitivity (AES) on the relationship between job complexity T1 and employee task proactivity T2 (Study 2).

**Figure 3:**
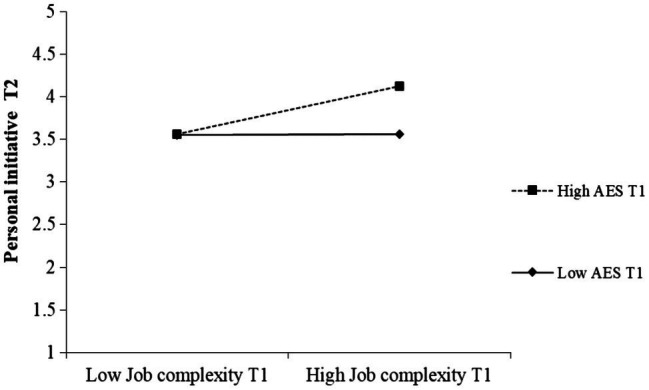
The moderating effect of AES on the relationship between job complexity T1 and employee personal initiative T2 (Study 2).

AES T1 was found to moderate the relationship between job complexity T1 and task proactivity T2 (*β* = 0.22, *p* = 0.015) as well as personal initiative T2 (*β* = 0.18, *p* = 0.036). A simple slopes test revealed that job complexity T1 was positively related to task proactivity at T2 if AES T1 was high (1 *SD* above the mean; *B* = 0.34, *SE =* 0.125, *t* = 2.759, *p* = 0.007), but unrelated when AES T1 was low (1 *SD* below the mean; *B* = −0.08, *SE* = 0.12, *t* = −0.648, *p* = 0.518). Similarly, for those employees who scored high on AES T1, job complexity T1 was positively related to personal initiative at T2 (*B* = 0.25, *SE* = 0.08, *t* = 3.324, *p* = 0.001). For employees who rated their AES T1 to be lower (1 *SD* below the mean), job complexity T1 showed no significant association with personal initiative measured one month later (*B* = 0.03, *SE* = 0.00, *t* = 0.450, *p* = 0.653). The significant interaction effects are depicted in Figures 2 and 3. These results support Hypothesis 3b that the relationship between job complexity and proactive work behavior is stronger for employees high in AES than for employees low in AES. All hypotheses were tested with and without controlling for gender and age as covariates. Both types of analyses led to the same conclusions.

## General Discussion

SPS has gained considerable societal attention in recent years, and research is steadily growing (Greven et al., 2019). However, its potential influences in the work setting lack scientific evidence, and research on individual work behavior is specifically absent (for some exceptions, see Andresen et al., 2018; Vander Elst et al., 2019). The objectives of this research were to investigate the two roles of SPS: (a) as a multidimensional personal characteristic and predictor of employee proactivity and (b) as a boundary condition in the way employees respond to job complexity with regards to their proactivity. Employee proactivity is an important form of behavior in today’s increasingly dynamic work context (Grant and Ashford, 2008).

The present study contributes to the literature by showing that the three SPS dimensions relate to employees’ proactive work behavior in different ways and that SPS can partly explain for whom complex jobs may stimulate proactivity. The study could not provide evidence for the assumption of EOE and LST—the two SPS dimensions associated with heightened activity in the BIS (Sobocko and Zelenski, 2015; Pluess et al., 2018; Lionetti et al., 2019)—inhibiting approach behavior, such as work proactivity. Although LST was negatively related to personal initiative in Study 2, this relationship could not be found for task proactivity as the second indicator of proactive work behavior, could not be replicated in Study 1, and did not exist for EOE.

Based on the P–E fit approach and previous research on EOE and LST as vulnerability factors of individual outcomes, it was further argued that the relationship between job complexity and employees’ proactive work behavior is less strong or negative for individuals reporting high levels of EOE and LST compared with those low in EOE and LSF. This assumption could not be supported. For individuals high in EOE and LST, job complexity does not seem to indicate a misfit, resulting in lower proactive work behavior one month later. Overall, the data do not support the role of EOE and LST as vulnerability factors of proactive work behavior. Hence, research showing that EOE and LST are related to adverse well-being outcomes (e.g., Evers et al., 2008; Vander Elst et al., 2019) does not necessarily apply to behavioral outcomes or proactive forms of work behavior, specifically (Harms et al., 2019).

AES was proposed to act as a supporting factor for proactive work behavior due to its relationship with heightened sensitivity of the BAS (Gray, 1991; Lionetti et al., 2019), indicating high appetitive motivation and the urge to engage in approach behavior, which should stimulate proactivity. Positive relationships were found between AES and employees’ proactive behavior across both studies. Yet, in Study 2, this relationship appeared for personal initiative but not for employees’ task proactivity. It can only be speculated why this finding emerged. One possible reason could be that Study 2, but not Study 1, was conducted during the COVID-19 pandemic and thus, different conditions and circumstances prevailed for the two studies. People’s awareness of and openness to positive environmental stimuli did not shape individual differences in their initiative to change their core work tasks, but it predicted their self-starting and future-oriented behavior in a broader sense (e.g., their searching for solutions and realizing ideas in the work context beyond their core works tasks; Frese et al., 1997; Frese and Fay, 2001). This assumption warrants further investigation. Overall, heterogeneity across circumstances, time points (Yarkoni, 2022) as well as differences in the effects for diverse indicators of proactive work behavior (Parker and Collins, 2010) should be considered in future research.

In addition, the present study found significant interaction effects between job complexity and AES on both indicators of employee proactive work behavior. The main effect of job complexity on proactivity was positive and significant for personal initiative only, whereas job complexity was positively related to both indicators of proactivity in employees with high AES but unrelated if AES was low. This finding supports the view that relationships between job complexity and proactive work behaviors are more complex and, in line with the P–E fit approach (Kristof-Brown et al., 2005), personality factors should be considered as key moderators. This finding also supports the view that for certain sensitive people (i.e., those with high awareness of and openness to positive stimuli in their surroundings), sensitivity does not necessarily have to be debilitating. Rather, following the perspective of vantage sensitivity (Pluess, 2017), when exposed to an enriching (e.g., complex) environment, people with high AES benefit in terms of their behavioral outcomes. Notably, however, the SPS dimensions could only explain a small percentage of variance in employee proactivity. This is not surprising given that SPS as a personality trait acts as a *distal* antecedent of proactive work behavior (Parker et al., 2010). Distal antecedents trigger behavior through more proximal proactive motivational states (i.e., being energized to, having a reason to, and having the confidence to show initiative and implement changes at work). These proximal states were not examined in the current study.

Across both studies, support was found for the three-dimensional nature of SPS as the most widely supported psychometrical solution (Smolewska et al., 2006; Vander Elst et al., 2019). EOE and LST were moderately and significantly related, whereas AES was unrelated to both EOE and LST. The three dimensions showed differential relationships with the other variables in the model, thus supporting the conceptual differences and the treatment of SPS on the dimension level (Greven et al., 2019). However, the psychometric characteristics, at least in Study 1, were not ideal. The reliability of the five-item measure of AES was low (alpha < 0.70), an issue that was also reported in previous research (e.g., Liss et al., 2008; Sobocko and Zelenski, 2015; Yano and Oishi, 2018). Although better than alternative models, the fit of the three-dimensional model was barely acceptable in Study 1; however, the fit was better in Study 2. The problematic factor structure of SPS measures is widely discussed (e.g., Greven et al., 2019; Vander Elst et al., 2019; Hellwig and Roth, 2021). Efforts should be made to revise and improve SPS measures further so that psychometrically sound measures are available to test hypotheses derived from theory.

Finally, the mean scores of the three SPS dimensions differed slightly between the studies, particularly for LST with means lower in Study 2. Differences in the characteristics of the samples may (partly) explain these divergent findings in the means. While Study 2 is based on a sample of employees with most of them residing in the United Kingdom, Study 1 includes an international sample of employees coming from various English-speaking and non-English-speaking countries. Cultural differences in the understanding of and sensitivity to certain SPS items have not yet been studied extensively (Listou Grimen and Diseth, 2016; Pluess et al., 2018; Greven et al., 2019). Nevertheless, existing evidence shows that individuals from different countries and backgrounds tend to differ in these aspects (see Greven et al., 2019, who refer to data showing that British participants score lower than Belgian individuals on certain SPS items). However, the participants might not only have varied in their cultural backgrounds but also in their language proficiency. Study 2 participants included native English speakers, whereas Study 1 participants were required to be fluent in English and able to complete the English surveys. Linguistic problems in terms of misunderstandings and misinterpretations might have occurred in Study 1. Potentially lower language proficiency might have also influenced the psychometric issues of the SPS scale in Study 1. Research suggests that non-native speakers are more likely to provide data of lower quality in survey studies (Lenzner et al., 2010; Wenz et al., 2021).

### Limitations and Implications for Future Research

Several limitations of the present study should be noted. The results are based on studies with a two-wave design, which is an improvement to existing, mostly cross-sectional research on SPS in the work context. However, although the temporal separation of predictor and outcome variables reduces the likelihood of common method bias (Podsakoff et al., 2003), the data are correlational in nature, so unambiguous conclusions about the direction of causality cannot be drawn. Reverse causation may also be possible such that engaging in proactive work behavior may increase employees’ job complexity (Frese et al., 2007). Moreover, the statistical power to detect interaction effects in Study 2 was low, increasing the likelihood of false-negative findings (Cohen, 1992).

The present study presents an interesting point of departure for the role of SPS in employee proactivity that calls for more research. First, the current perspective is limited in the sense that it is insufficient to focus on distal personality variables and perceptions of job complexity as job demands as the sole motivators of proactive work behavior. Future research is needed to better understand individuals’ motivational states that are more proximal to goals and action (Parker et al., 2010). In a related vein, extending Study 2, future research could study the underlying mechanisms of the interaction between job complexity and the different SPS dimensions with regard to proactive work behavior in a larger sample. Cognitive appraisal may play an important role in determining how employees perceive and react to job complexity depending on their level of SPS (Lazarus and Folkman, 1984). For instance, individuals with high AES, who are more open to positive experiences and new ideas, appraise complex tasks as being more challenging and motivating, and because of their approach orientation they are more likely to engage in proactive behavior to improve situations (Espedido and Searle, 2020).

Second, this study investigated the interplay between job complexity as a work-related demand and SPS as a personality characteristic. According to the demand–control–person model (Rubino et al., 2012), this perspective could be extended by examining the role of job resources (e.g., support, job autonomy, task routinization) as another boundary condition that may improve predictions about individual behavioral reactions to job demands, such as job complexity (Vander Elst et al., 2019). For instance, individuals high in EOE and LST might be less vulnerable to the negative effects of complexity when they experience a supporting organizational climate (Hong et al., 2016) or when routinized work tasks are implemented (Ohly et al., 2006), an assumption that might help to explain the non-significant two-way interaction effects found in the current study.

Moreover, other personality characteristics might play a role in explaining the missing moderating effects of EOE and LST. If employees have strong aspirations of controlling their environment or a generally strong proactive personality (Frese et al., 2007) along with their tendency of being easily overwhelmed and feeling aroused by internal or external stimuli, their proactive work behavior might not suffer. Testing these three-way interactions of demands, personality traits, and other contextual and personal factors might provide a more comprehensive perspective on this issue. Finally, because SPS is highly correlated with well-established personality characteristics, such as neuroticism, introversion, and openness to experience (Greven et al., 2019; Lionetti et al., 2019; Hellwig and Roth, 2021), future research on relationships between SPS and employee work behavior should explore its incremental validity by investigating whether the SPS dimensions predict employee outcomes above and beyond the Big Five traits.

### Practical Implications and Conclusion

Although SPS is generally understood as a vulnerability factor that may increase the risk for the development of mental problems (Aron et al., 2012), the current findings propose that SPS cannot be seen as a “weak” personality characteristic when it comes to predicting employees’ proactive behaviors (Harms et al., 2019). Individuals high in EOE and LST do not seem to show lower proactive behaviors and do not differ from those low in these dimensions when dealing with complexity at work. Employees high in AES tend to engage in proactive work behavior to a greater extent and benefit more from job complexity than those low in AES. Further research is needed before profound practical implications can be inferred, but the current findings suggest that employers can stimulate proactivity in some of their more sensitive employees (i.e., those high in AES) by providing them with more complex work tasks. Generally, more awareness of the positive aspects of SPS in the organizational context can be gained based on these findings.

## Data Availability Statement

The raw data supporting the conclusions of this article will be made available by the authors, without undue reservation.

## Ethics Statement

The two studies were reviewed and approved by the Research Ethics Committee of the Department of Psychology, University of Groningen, the Netherlands. The participants provided their written informed consent to participate in this study.

## Author Contributions

The author confirms being the sole contributor of this work and has approved it for publication.

## Conflict of Interest

The author declares that the research was conducted in the absence of any commercial or financial relationships that could be construed as a potential conflict of interest.

## Publisher’s Note

All claims expressed in this article are solely those of the authors and do not necessarily represent those of their affiliated organizations, or those of the publisher, the editors and the reviewers. Any product that may be evaluated in this article, or claim that may be made by its manufacturer, is not guaranteed or endorsed by the publisher.
